# Identifying spatial and temporal organization in the circadian clock (Commentary on Pauls *et al*.)

**DOI:** 10.1111/ejn.12670

**Published:** 2014-08-03

**Authors:** Hugh D Piggins

**Affiliations:** Faculty of Life Sciences, University of ManchesterOxford Road, Manchester, M13 9PT, UK

“The conditions you need to be a good goalkeeper are exactly the same condition you need to be a good sculptor. You must have a very good connection in both professions, with time and space.”

Eduardo Chillida

To organize circadian (∼24 h) rhythms in physiology and behavior, the brain's master circadian clock in the suprachiasmatic nuclei (SCN) must, like a proficient goalkeeper or sculptor, coordinate and connect its signals in both time and space. Individual SCN neurons contain the intracellular molecular clockworks, but these several thousand cell autonomous oscillators require intercellular communication to synchronize their timekeeping. How the SCN achieves the integration of intracellular and intercellular signals across its anatomical structure is far from understood. The current study by Pauls *et al*. (2014) presents new analytical tools to interrogate the spatiotemporal organization of the SCN.

Unlike many brain structures, the SCN sustains its function *ex vivo* in culture. Indeed, SCN explants prepared from rodents bearing bioluminescent reporters of the molecular clock (such as PER2::Luc mice) sustain ∼24 h rhythms in bioluminescence. Although photovideomicroscopy recordings from such explants are visually appealing, they present considerable challenges for analysis as frame-by-frame tracking of the cycles of gene expression in individual SCN cells is both time- and labour-intensive. Here, the authors develop a fast, automated approach that appears to circumvent such problems. To do this, the authors took photovideomicroscopy recordings made from neonatal PER2::Luc SCN explants and applied image processing followed by a cluster and spectral cluster analysis, based on similarity of luminescence signals, to generate quantifiable maps of PER2 expression throughout entire SCN slices. They then show that their automated procedure generates results consistent with previous manual assessment of cellular rhythms in these recordings. This is reassuring as it indicates that both approaches yield similar outcomes. However, the automated procedure is much faster and avoids the inherent undersampling of SCN regions that often arises in manual analysis based on putative single cells. Subsequently they determined that there is a marked spatial organization within the SCN such that proximal regions of the SCN slice can be resolved into ‘clusters’ of similar bioluminescence signal that persist over several days of recording. Intriguingly, when circadian time scales were filtered out, this spatial organization remained. Spectral embedding analysis showed that there was a strong relationship between spatial and temporal organization. Unsurprisingly, in SCN recordings from animals lacking a functional molecular clock, there was no temporal organization, but interestingly, a spatial organization was still detected. In some recordings from the SCN of mice lacking a key intercellular signal, some temporal organization was detected, whereas spatial organization was lacking. In summary, the current study reports a quick, efficient and sophisticated methodology to analyse the spatial and temporal organization of gene/protein expression in SCN tissue explants. This approach reveals the important inter-relationship between spatial and temporal influences and also shows how the two are separable when intracellular or intercellular signals are absent. One caveat is that it is unclear whether the spatial organization uncovered by such analysis identifies functionally distinct areas of the SCN or simply reflects the differential density of cells expressing clock genes. No doubt subsequent iterations of this approach can integrate this information.

## References

[b1] Pauls S, Foley NC, Foley DK, LeSauter J, Hastings MH, Maywood ES, Silver R (2014). Differential contributions of intra-cellular and inter-cellular mechanisms to the spatial and temporal architecture of the suprachiasmatic nucleus circadian circuitry in wild-type, cryptochrome-null and vasoactive intestinal peptide receptor 2-null mutant mice. Eur. J. Neurosci.

